# Correction: Organisational kindness and compassion: what are the barriers, enablers and outcomes for clients and stakeholders?

**DOI:** 10.1371/journal.pone.0337341

**Published:** 2025-11-19

**Authors:** The PLOS One Staff

In the Introduction, there is an error in the first sentence of the first paragraph. The correct sentence is: Unkindness is defined as “not treating someone well, or not considering someone’s feelings” [1].

The publisher apologizes for the error.
