# Supplementary material for: Organisational kindness and compassion: what are the barriers, enablers and outcomes for clients and stakeholders?
Source: PLoS One. 2025 Jun 25;20(6):e0312450. doi: 10.1371/journal.pone.0312450 (PMC12192139; doi:10.1371/journal.pone.0312450)
Supplement: Attachment — Submitted filename: Response to Reviewers.docx [file pone.0312450.s002.docx]

Reviewer #1:

| **Reviewer’s comment** | **Authors’ response** |
| --- | --- |
| Abstract: The abstract is well done but it appears to be too long. I also stress that the abstract should summarize the introduction. | The abstract has been shortened in length while also now incorporating key elements of the introduction.  See page 1 |
| Introduction: First, the introduction should summarize the whole paper by (a) stating the problem to be addressed and justifying the value of doing so, (b) specifying how the problem is or was addressed (materials and methods), (c) outlining the results and their implications, and (d) drawing a conclusion and what it recommends for policy and further research. Some elements of these are there but are not all expressed succinctly, and they should be. | The introduction has been revised as suggested.   a) See page 7 paragraph 2 b) See page 8 paragraph 1 c) Results are details in the subsequent section d) See page 8 paragraph 1 |
| Second, since there is no formal literature review, it would be informative if the authors stated what is missing from the existing literature and what this paper contributes to what is already known. In the Paragraph starting with “Stepping back …” (p. 4), there is a need to provide references specific to “organizational kindness and compassion.” | The paragraph in question has been revised with references added that are specific to organisational kindness and compassion.  See page 5 paragraph 2 |
| Third, on p. 5, “kindness” is not well characterized. For example, is kindness a direct or incidental function of policy? How is kindness and compassion measured? In private (for-profit) organizations, do “external clients and stakeholders” include investors or not? | Each of these three questioned raised by the reviewer are now explicitly addressed.  See page 5 paragraph 2, page 6 paragraph 2 and the last paragraph of page 7 |
| Method: This section appears well-done. However, I wished each stage was better motivated. How did the authors determine “potentially relevant studies” and how many of these did the scoping turn up? Stage 4 (p. 8) talks about “a charting framework” what is that (describe) and how was it “developed”? There are also undefined acronyms like DM, JSM, JNS, and NVivo – what are these, and why should the reader accept the soundness of all these? | The method section states, “Potentially relevant studies were then identified by applying the search terms to the following databases: Scopus; ProQuest Central; PsycINFO; Business Source Ultimate via EBSCO.”  The *results section* has now been updated to state that 489 potentially relevant publications were derived from the scholarly database searches.  See page 11 paragraph 1  The Method section (stage 4 - extracting data from relevant studies) has been updated to better explain the charting framework.  See page 10, paragraph 1.  The use of researcher initials has been explained in terms of transparently identifying which researcher conducted which research-related task (while maintaining the anonymity required for this blinded manuscript review process). (See page 8 paragraph 2).  NVivo is now clearly defined as software that is designed to support researchers in conducting qualitative data analysis. (See page 9 paragraph 2) |
| Results: The results are reasonable. However, occasionally, the paper appears not to distinguish clearly enough the difference between organizational kindness and compassion and the kind and compassionate actions of individuals. If Microsoft CEO donates to an orphanage in Mozambique, is he or Microsoft who is kind and compassionate? | The results section has been updated throughout to clearly differentiate between instances of organisational kindness and compassion and the kind and compassionate actions of *individuals*.  See page 15 paragraph 1, paragraph 3, page 16 paragraph 2, page 17, paragraph 1. |
| Discussion: I expected more examples than just education and health care to minimize the fallacy of composition. | The fact that this literature focused mainly on healthcare and education policy areas is now explicitly reflected upon in our Discussion and Conclusion (i.e., future research directions) sections. See page 19 paragraph 2, See page 24 paragraph 2. |
| Limitations: Well-taken. | The authors’ appreciate this recognition. |
| Conclusion: This one is the strongest section of the paper in terms of presentation. The only question it raises is why the conclusion does not have any implications for future research. Should the reader conclude this case is open and shut? | We have added implications for future research directions.  See page 24 paragraph 2. |

Reviewer #2:

| **Reviewer’s comment** | **Authors’ response** |
| --- | --- |
| 1. Support for Claims: The claims regarding the impact of kindness and the listed factors need to be better supported by data. The extent to which these factors hamper or facilitate kindness and their impact on stakeholders should be rigorously displayed. Quantitative analysis from the literature review should be presented more clearly to substantiate these findings. | As noted in Table 1, only one quantitative study was included among our publications. Our limitations section and conclusion (i.e., future research directions) have been revised to reflect this. See page 23 paragraph 1, See page 24 paragraph 2 |
| 2. Scope of Literature:  The available literature is limited and mainly focused on healthcare and education. There appears to be a gap in sources specifically addressing the welfare area. This limitation should be explicitly acknowledged in relevant sections of the paper. | A literature focus mainly on healthcare and education policy areas is now captured in the discussion and conclusion (i.e., future research directions) sections. See page 19, paragraph 2, See page 24 paragraph 2 |
| 3. Content Revision and Organization:  The remaining paragraphs require content revision and re-organization to enhance readability. Topics should be consolidated rather than scattered across different paragraphs. | Content have been revised to ensure that each paragraph is confined to addressing a specific subject as clearly identified in a topic sentence. Revisions have also supported the smooth and logical flow of information throughout. |
| 4. Relevance and Completeness:  It might be worth mentioning the reforms prompted by the negative consequences of the Robodebt scandal in the management of welfare systems and the implementation of automated decisions in recent years, for better completeness. | Policy reforms following the Royal Commission into the Robodebt scheme are now detailed.  See page 20, paragraph 2. |
| 1. Theoretical Frameworks:  o Apply theoretical frameworks from organizational behavior, compassion, and social welfare to structure analysis and draw meaningful conclusions. | The authors have included and applied theoretical frameworks from organisational behaviour, compassion, and social welfare frameworks as suggested (e.g., micro-analysis, and systems theory).  See page 19, paragraph 2.  See page 20, paragraph 2. |
| 2. Cross-Sector Analysis:  Draw parallels from existing research in healthcare and education, explaining how findings might be relevant or transferable to the welfare sector. | The discussion has been updated to include cross-sector analysis (e.g., our call for studies to examine possible opportunities to advance a micro-level focus as prominent in the healthcare sector to better support welfare sector recipients and in particular external stakeholders with disability).  See page 19, paragraph 2. |
| 3. Future Research:  Emphasize the need for future research focused specifically on organizational kindness in the welfare sector, calling for further studies by other researchers and policymakers. This could include broadening the literature review scope to include related fields such as social work, public policy, and community health, which may offer insights applicable to the welfare context. Conduct primary research within the welfare area, including surveys, interviews, or case studies with stakeholders, to gather specific data on organizational kindness, for a comprehensive understanding of organizational kindness in the welfare sector. | We appreciate the suggestions provided and these specific future research directions have been included in our revised conclusion.  See page 24 paragraph 2. |
| 1. Abstract:  o Mention the Australian policy (Robodebt) given its relevance to the research. This will provide better context and highlight the significance of the study from the outset. | The abstract has been revised to mention Robodebt policy as suggested.  See page 1. |
| 2. Introduction:  Move the New Zealand example to the introduction and reference it when discussing barriers instead of waiting for the discussion section to introduce it. This helps to set the stage and provides a comparative context early in the paper | Revised as suggested.  See page 7, paragraph 1. |
| 3. Conceptual Clarity:  o Distinguish between kindness and compassion: while connected, they are distinct concepts. Consider listing compassion among the enablers. This would also necessitate a revision of the research title.  o Provide detailed definitions of key terms such as "inauthentic behaviors," "respect," and "professional behavior", acknowledging their potential for varied interpretations. | The distinction between kindness and compassion has been explicitly made as suggested.  See page 5, paragraph 2.  Definitions and qualifiers about interpretations have been included throughout as suggested. Note that we make no reference to professional behaviour throughout the manuscript. |
| 4. Data Presentation:  Enhance the presentation of quantitative findings. Clearly display how factors identified from the literature review impact organizational kindness and the resulting effects on stakeholders. | As noted in Table 1, only one quantitative study was included among our publications.  See page 11, paragraph 1.  Our limitations section and conclusion (i.e., future research directions) have been revised to reflect this. |
| 5. Revisions for Readability:  Reorganize content to ensure each topic is discussed in one place. Avoid scattering related information across different paragraphs.  o Ensure a logical flow of ideas from the introduction through to the conclusion. | Content have been revised to ensure that each paragraph is confined to addressing a specific subject as identified in a topic sentence.  The information flows logically throughout (i.e., introduction, method, results, discussion, limitations, and conclusion). This logical flow is maintained in our discussion section: overall findings; literature focus on healthcare and educational organisations; moving beyond healthcare and education policy settings – welfare; the kindness contagion effect; trust and kindness shown towards external organisational stakeholders; and kindness/compassion related risks to organisational leaders. |
| 6. Discussion:  Currently, the discussion is scattered, which hampers the reader's ability to get an overview of the key points. Use this section to interpret the results, linking them back to the research questions and existing literature.  o Discuss the practical implications for organizational policy and practice, especially in light of the Robodebt scandal.  o Provide clear recommendations based on the results.  o Identify areas for future research to address gaps and build on the findings of the current study. | Content have been revised to ensure that each paragraph is confined to addressing a specific subject as identified in a topic sentence and that information flows in a smooth and logical way.  The discussion now clearly links results back to our respective research aims.  See page 18, paragraph 3.  See page 19, paragraph 2.  Practical implications from the Robodebt royal commission and examples of its key recommendations have been included in the Discussion section.  See page 20, paragraph 2.  Areas for future research to address gaps and to build on the findings of our study are made explicit in the Discussion and Conclusion sections.  See page 19, paragraph 2.  See page 24, paragraph 2. |
| 7. Policy Reforms:  Mention the reforms prompted by the negative consequences of the Robodebt scandal in the discussion. This will provide a more comprehensive view of the changes in welfare systems and the management of automated decisions. | Key policy reforms following the Royal Commission into the Robodebt Scheme are now mentioned in our Discussion section.  See page 20, paragraph 2. |
| 8. Conclusion:  Summarize the key findings and their implications succinctly.  Reinforce the importance of organizational kindness and compassion in the context of social welfare.  Highlight the need for further research to explore these concepts more deeply in other sectors beyond healthcare and education. | The Conclusion section has been revised as suggested. |

* Note paragraph and page numbers align with the clean version of our manuscript.
