# Supplementary material for: Organisational kindness and compassion: what are the barriers, enablers and outcomes for clients and stakeholders?
Source: PLoS One. 2025 Jun 25;20(6):e0312450. doi: 10.1371/journal.pone.0312450 (PMC12192139; doi:10.1371/journal.pone.0312450)
Supplement: Attachment — Submitted filename: Additional Editor Comments and Authors Responses.docx [file pone.0312450.s003.docx]

| **Additional Editor’s Comments** | **Authors’ Response** |
| --- | --- |
| 1. Of these we identified a total of 25 publications that met our inclusion (see Figure 1 for PRISMA diagram). - did you mean "inclusion criteria"? | Edited as suggested. |
| 2. Reflecting upon strong criticisms received by New Zealand’s former Prime Minister Jacinda Ardern (61), we note, however, that these kinds of negative and unjustified stakeholder responses to compassionate leadership are not a given. - not fully understandable | The sentence has been revised to improve clarity as follows:  Reflecting upon strong criticisms received by New Zealand’s former Prime Minister Jacinda Ardern (62), we note, however, that these kinds of negative and unjustified stakeholder responses to compassionate leadership are not guaranteed to occur. |
| 3. The findings were mainly limited to qualitative studies, and impacted by small sample sizes. And the possibility of selection bias with study participants not randomly selected. - maybe not to start the second sentence with "And"? | “And” has been removed as suggested. The sentence now starts with, We also note the possibility of selection bias… |
| 4. These results provide a frame for considering the results of organisational compassion and kindness on external stakeholders, and the factors that limit or make this possible. - I am not sure what you meant by this sentence. | The sentence has been revised to improve clarity as follows:  These results provide a frame for considering organisational compassion and kindness involving external stakeholders, and the factors that limit or make this possible. |
