# Supplementary material for: Organisational kindness and compassion: what are the barriers, enablers and outcomes for clients and stakeholders?
Source: PLoS One. 2025 Jun 25;20(6):e0312450. doi: 10.1371/journal.pone.0312450 (PMC12192139; doi:10.1371/journal.pone.0312450)
Supplement: Attachment — Submitted filename: Response to Reviewers1.docx [file pone.0312450.s005.docx]

Dear Editor,

Thank you for the opportunity to improve our manuscript. We have carefully considered each of the points raised by Reviewer 3 (noting your advice of “no amendments are required in response to reviewer 4’s comments”) with our responses provided in Appendix A, Table 1 below. Our responses are reflected in the following two uploaded files as part of our submission: a) Revised Manuscript with Track Changes; and b) Manuscript. We very much look forward to hearing of a final decision on our submitted revisions.

Kind regards,

Dr Damian Mellifont (corresponding author)

Email: [damian.mellifont@sydney.edu.au](mailto:damian.mellifont@sydney.edu.au)

Appendix A.

Table 1. Authors’ responses to Reviewer 3 Points.

| Academic Editor’s Point | Authors’ response |
| --- | --- |
| “- I am uncomfortable with the reification (see Oxford Reference entry) -- namely that an organization can be kind or express kindness. Rather, it is more accurate to say that it is the people who work there -- at all levels of decision-making and power -- who behave in kind ways and/or follow policies and procedures that direct them to be kind? It is the policies that have been produced by people that enact/constrain kindness. I think as long as the authors write a sentence to acknowledge that they are reifying organizations (and even argue in favor of doing so), it will read as a more scholarly paper.” | The acknowledgement sentence has been added as suggested (see page 8). |
| “- has it been established what the examples, typologies, themes, etc. of organizational kindness are? this almost seems to be a first step in the authors' research, but is missing from the paper.” | We now map out this more clearly and provide a definition of organisational kindness. We note shared constructions of organisational kindness is a persisting, social and reflective process, but that organisational kindness is also reflected in a range of practical examples within the literature (see changes on pages, 5, 6, 8). |
| “ - would suggest that the authors explain the nuanced differences/similarities between kindness and compassion that make these terms interchangeable in the study” | The nuances between kindness and compassion that make these terms interchangeable in the study are further explained as suggested (see pages 5, 6, 8). |
| “ - suggest a table with the themes, and perhaps a concept map or diagram? how do they relate with each other?” | Figure 2 has been added as a concept map (PACE verified) as suggested (pages 12 and 13). |
